# Investigating Smartphone-Based Sensing Features for Depression Severity Prediction: Observation Study

**DOI:** 10.2196/55308

**Published:** 2025-01-30

**Authors:** Yannik Terhorst, Eva-Maria Messner, Kennedy Opoku Asare, Christian Montag, Christopher Kannen, Harald Baumeister

**Affiliations:** 1 Department of Clinical Psychology and Psychotherapy Institute of Psychology and Education Ulm University Ulm Germany; 2 Department of Psychology LMU Munich Munich Germany; 3 German Center for Mental Health (DZPG), Partner Site Munich-Augsburg Munich Germany; 4 Center for Ubiquitous Computing University of Oulu Oulu Finland; 5 Department of Molecular Psychology Institute of Psychology and Education Ulm University Ulm Germany

**Keywords:** smart sensing, digital phenotyping, depression, observation study, smartphone, mHealth, mobile health, app, mental health, symptoms, assessments

## Abstract

**Background:**

Unobtrusively collected objective sensor data from everyday devices like smartphones provide a novel paradigm to infer mental health symptoms. This process, called smart sensing, allows a fine-grained assessment of various features (eg, time spent at home based on the GPS sensor). Based on its prevalence and impact, depression is a promising target for smart sensing. However, currently, it is unclear which sensor-based features should be used in depression severity prediction and if they hold an incremental benefit over established fine-grained assessments like the ecological momentary assessment (EMA).

**Objective:**

The aim of this study was to investigate various features based on the smartphone screen, app usage, and call sensor alongside EMA to infer depression severity. Bivariate, cluster-wise, and cluster-combined analyses were conducted to determine the incremental benefit of smart sensing features compared to each other and EMA in parsimonious regression models for depression severity.

**Methods:**

In this exploratory observational study, participants were recruited from the general population. Participants needed to be 18 years of age, provide written informed consent, and own an Android-based smartphone. Sensor data and EMA were collected via the INSIGHTS app. Depression severity was assessed using the 8-item Patient Health Questionnaire. Missing data were handled by multiple imputations. Correlation analyses were conducted for bivariate associations; stepwise linear regression analyses were used to find the best prediction models for depression severity. Models were compared by adjusted *R*^2^. All analyses were pooled across the imputed datasets according to Rubin’s rule.

**Results:**

A total of 107 participants were included in the study. Ages ranged from 18 to 56 (mean 22.81, SD 7.32) years, and 78% of the participants identified as female. Depression severity was subclinical on average (mean 5.82, SD 4.44; Patient Health Questionnaire score ≥10: 18.7%). Small to medium correlations were found for depression severity and EMA (eg, valence: *r*=–0.55, 95% CI –0.67 to –0.41), and there were small correlations with sensing features (eg, screen duration: *r*=0.37, 95% CI 0.20 to 0.53). EMA features could explain 35.28% (95% CI 20.73% to 49.64%) of variance and sensing features (adjusted *R*^2^=20.45%, 95% CI 7.81% to 35.59%). The best regression model contained EMA and sensing features (*R*^2^=45.15%, 95% CI 30.39% to 58.53%).

**Conclusions:**

Our findings underline the potential of smart sensing and EMA to infer depression severity as isolated paradigms and when combined. Although these could become important parts of clinical decision support systems for depression diagnostics and treatment in the future, confirmatory studies are needed before they can be applied to routine care. Furthermore, privacy, ethical, and acceptance issues need to be addressed.

## Introduction

Depression is associated with high personal burden, impaired social participation and functioning, increased mortality, and high economic burden [1-3]. In 2020, depression was one of the leading causes of disability-adjusted life years worldwide (49.4 million; 95% CI 33.6-68.7) and is expected to be the leading cause by 2030 [1,3,4]. Despite its severity and the existence of effective treatments for depression [5-7], only 41.8% of people with major depressive disorder (MDD) receive any mental health services, and less than 30% of people with MDD receive adequate treatment [1,8]. Although several barriers contribute to this issue (eg, availability, accessibility, and acceptability of treatment) [1,8,9], a fundamental prerequisite to any health services is a timely and accurate diagnosis of MDD, or more generally, the assessment of depression severity to initiate an informed treatment process [10-14].

Well-established assessments like structured clinical interviews are often not feasible to be conducted in primary care or for preventive screening purposes (eg, due to time pressure or limited availability of qualified personnel) [10-13,15]. Furthermore, if they are implemented, they typically take place at a fixed time point and assess symptoms retrospectively, which makes them subject to several biases (eg, recall bias) and unable to assess the dynamic and fluctuating nature of mental health [16-18]. Hence, novel diagnostic approaches, which can be easily integrated into daily living to monitor depression severity with high ecological validity, could make an important contribution to improve and augment current diagnostic procedures for depression—particularly if they provide fine-grained insights into mental health symptomology (eg, on daily level). Given the omnipresence of smartphones in everyday life, the unobtrusive collection of objective sensor data (eg, total haversine distance between tracked GPS coordinates, social contacts, screen, and app usage) might be a promising step toward improved diagnoses [19-21]. This process is also referred to as smart sensing (also known as mobile sensing or digital phenotyping) in the context of depression [19-22]. Applications of smart sensing range from supporting initial diagnosis to integration during treatment (eg, just-in-time-adaptive interventions) or after treatment (eg, just-in-time interventions in relapse prevention) [19,23].

In the field of depression so far, many studies followed a classificatory approach classifying persons as depressed or not depressed (eg, based on the 8-item Patient Health Questionnaire [PHQ-8] cutoff ≥10) [24-28]. For instance, a first meta-analysis on supervised machine learning models to predict depression status based on wearable data reported an average accuracy of 0.89, 95% CI 0.83-0.93 (sensitivity: 0.87, 95% CI 0.79-0.92; specificity: 0.93, 95% CI 0.87-0.97) [24]. However, such a classificatory understanding of depression seems questionable when looking at (1) the poor agreement of domain experts in the diagnosis of depression [29,30], (2) the heterogeneity of symptom networks in depressed patients [31,32], and (3) the profound evidence for depression and general psychopathology being a continuous spectrum [29,33]. Hence, studies operationalizing depression as a continuous spectrum and understanding the prediction as a regression instead of a classification problem are highly needed in the field.

For example, for GPS features (eg, total distance, number of significant places), a meta-analysis shows that robust correlations between sensing features and depression severity as continuous dimension exist (eg, distance: *r*=–0.25, 95% CI –0.29 to –0.21), time spent home: *r*=0.10 (95% CI 0-0.19), or normalized entropy: *r*=–0.17, 95% CI –0.29 to –0.04) [34]. Besides, initial studies highlight the potential of features obtained from the screen (eg, smartphone usage duration), app (app usage), and call (eg, number of incoming calls) sensors [35,36]. However, so far, analyses are often limited to bivariate correlations and do not extend to (1) the variance in depression severity, which can be explained by the features, and (2) the combination of multiple features and which incremental benefit they provide (eg, in explained variance) [35].

Against this background, this study aimed to extend the evidence for various sensor modalities collected via the smartphone (ie, screen features, app usage features, location or GPS features, and call features) by (1) investigating bivariate correlations, (2) exploring the explained variance and incremental benefit of features in cluster-wise regression models (eg, limited to location features), and (3) cluster-combined regression models. Accordingly, the following research questions will be answered:

1. Which bivariate correlations are present between depression severity and (a) screen, (b) app, (c) location, and (d) call features?

2. How much variance in depression severity can be explained in parsimonious cluster-wise regression analyses (eg, limited to location features)?

3. How much variance in depression severity can be explained by the best cluster-combined regression model?

Besides, we wanted to compare the unobtrusively and objectively collected sensor features against features based on ecological momentary assessments (EMA; eg, average valence, average arousal), which, similarly to sensor data, provide a continuous assessment over time but require active input [18,35,37]. Therefore, we investigated the following questions:

4. Which bivariate correlations are present between depression severity and EMA features?

5. How much variance in depression severity can be explained in regression analyses using EMA features?

6. How much variance in depression severity can be explained in regression analyses using EMA and sensing features?

7. What is the difference in explained variance in depression severity between regression models limited to EMA features or sensor features compared to their combination?

## Methods

### Study Design

This study is an exploratory observation study investigating the associations between smart sensing features and depression severity. Accordingly, we followed the Strengthening the Reporting of Observational Studies in Epidemiology (STROBE) guidelines [38] (see Multimedia Appendix 1 for the STROBE checklist).

### Ethical Considerations

All procedures were assessed and approved by the local ethics committee of Ulm University, Germany (259/16-CL/bal). Informed consent was given by all participants, and participants were informed about their rights according to the European General Data Protection Regulation. Privacy and data security were assessed by Ulm University. Students of Ulm University participating in the study were eligible for study credits as an expense allowance. No other compensation was provided to participants.

### Study Population and Procedure

Aiming for a general population sample, participants (see eligibility criteria below) were recruited using an open recruitment strategy involving digital channels (eg, email lists and social media posts) and offline channels (eg, flyers at public institutions). Participants were informed about the purposes of and procedures in the study in an online survey and asked for their written informed consent. If given, they were instructed to install the smart sensing application of the INSIGHTS framework [39,40] after providing basic personal characteristics (ie, gender and age) in the online survey; afterwards, all data were collected via the app. See the assessment and features below for a description of all features and the technical framework paper for further details on the software [39].

### Eligibility Criteria for Participants and Episodes

Participants were included in the study if (1) informed consent was given and (2) participants were 18 years or older. (3) Due to the technical requirements of the sensing framework, participants were required to have a smartphone running on Android. Neither a diagnosis nor a minimum level of depression was required to be included in the study. Furthermore, we only included participants’ data in the analysis if (4) participants completed the depression severity questionnaire (PHQ-8; see details below) at least once. We structured the data of the participants in 14-day periods consisting of the depression questionnaire assessing the average depression severity in the last 14 days and the corresponding sensing data per day. The number of episodes varied across participants (mean 4.09, SD 3.55; range: 1-42). To avoid the biasing influence of participants being represented more often than others in the dataset and to maximize the data quality, we (5) included only the episode with the lowest amount of missingness per person. Missingness was determined across all days in the episode and features. In addition, (6) we excluded all participants with more than 50% missing data in EMA and sensing features during an episode to ensure missing data handling procedures (see below) were reliable [41,42]. Excluded participants, according to (6), did not significantly differ in age, gender, or depression symptomology from included participants, underlying the assumption that technical issues leading to the exclusion (eg, app not working) did not occur systematically (Multimedia Appendix 2).

### Assessment and Features

#### Overview

The assessment consisted of (1) self-report severity measures, (2) EMA, (3) smartphone screen features, (4) app usage features, (5) location features, and (6) call features. Following the validated procedures from previous studies, we used Python, Snakemake, and the Reproducible Analysis Pipeline for Data Streams (RAPIDS) framework in the data preprocessing and extraction pipeline of all smartphone features [28,43-45]. Smartphone features were calculated for each day and aggregated across the 14-day window (eg, average daily smartphone usage duration across 14 days). A summary of all included smartphone features can be found below.

#### Clinical Questions

We used the PHQ-8 for the assessment of depression severity. The PHQ-8 consists of 8 self-report items asking how often a symptom was present in the last 14 days (0=not at all to 3=nearly every day). Higher PHQ-8 sum scores indicate higher depression severity. The PHQ-8 is a reliable instrument (Cronbach α=.87, ω=.94) [46,47] (see Multimedia Appendix 3 for an overview of the PHQ-8 items).

#### EMA Questions

All items were rated from 0 (lowest imaginable) to 100 (highest imaginable). We assessed valence (higher values indicate positive affect), arousal (higher values indicate higher energy levels), and stress (higher values indicate higher stress levels) 3 times per day (morning, midday, and evening). Additionally, sleep quality (high values indicate good sleep) was assessed in the morning, and satisfaction (higher values indicate stronger satisfaction) with the quality of social interaction, the number of social interactions, activity, and nutrition was rated in the evening once per day. See [40] and Multimedia Appendix 4 for an overview of the EMA questions.

#### Screen Features

The screen sensor tracked the lock and unlock events of the smartphone. Based on this, we determined the number of usage sessions, duration of usage (sum, average, maximum), regularity index of all unlock episodes, entropy, and normalized entropy of unlock events [28,44].

#### App Features

For each app, we tracked the start and end of each usage session to calculate the count of all used apps, the mean duration of app usage per day, the regularity index of app usage, and the frequency entropy of app usage [44].

#### Location Features

Using the GPS sensor of the smartphone, we determined the total distance, logarithmic location variance, number of significant places, stay duration (average, maximum, SD, at top 1 location, at top 2 location, and at top 3 location), the ratio between the time spent at nonsignificant places to all clusters (percent of outlier time), location entropy, normalized location entropy, circadian movement, location routine index, number of location transitions, and moving to static ratio [44,48-54].

#### Call Features

Incoming, outgoing, and missed calls were tracked separately. For each of them, we tracked the *count* and distinct contacts. Furthermore, for incoming and outgoing calls, we calculated the duration (average, sum, and maximum) and entropy [28,44,54].

See Table 1 for an overview of the feature definition, interpretation, and references, providing an in-depth introduction to the background and calculations of the features. For further details on the Reproducible Analysis Pipeline for Data Streams framework, refer to its paper [44].

**Table 1:** Feature definition and interpretation.

Feature		Definition	Units and interpretation
**Ecological momentary assessment**			
	Valence	Affect ratings from users	0 (lowest) to 100 (highest); higher values indicate positive valence
	Arousal	Arousal ratings from users	0 (lowest) to 100 (highest); higher values indicate higher energy levels
	Stress	Stress ratings from users	0 (lowest) to 100 (highest); higher values indicate higher stress
	Sleep	Sleep quality ratings from users	0 (lowest) to 100 (highest); higher values indicate higher sleep quality
	Social quantity	Satisfaction with quality of social contacts reported by users	0 (lowest) to 100 (highest); higher values indicate higher satisfaction
	Social quality	Satisfaction with quality of social contacts reported by users	0 (lowest) to 100 (highest); higher values indicate higher satisfaction
	Nutrition	Nutrition ratings from users	0 (lowest) to 100 (highest); higher values indicate healthy nutrition
	Physical activity	Physical activity ratings from users	0 (lowest) to 100 (highest); higher values indicate higher intensity activity
**App usage**			
	App count	Number of app usage episodes in foreground	Count
	App duration	Duration of app usage episodes in foreground	Time in hours
	App frequency entropy	Entropy is a measurement of the degree of variability between the users’ behavior states. The frequency of use per app over a 24-hour period was used to calculate app frequency entropy. For calculation details, refer to [44,54]	Higher app frequency entropy reflects a more distributed usage of apps (ie, high variability). Lower values indicate that users used one app more often (ie, low variability)
	App regularity index	Regularity index captures the similarity of behavior between the same hours across different days. App regularity index refers to the similarity of the most frequently used app at the same hour across days. Calculations were based on [44,54,55]	Higher regularity index indicates a user used the same app at the same hours across days
**Screen usage**			
	Screen episode count	Number of screen unlock episodes	Count
	Screen duration	Duration of unlock episodes	Time in hours
	Screen regularity index	Regularity index captures the similarity of behavior between the same hours across different days. Screen regularity index refers to the similarity of the most frequent screen status (on or off) at the same hour across days. Refer to [44,55]	Higher regularity index indicates a user used their smartphone at the same hours across days
	Screen entropy	Entropy is a measurement of the degree of variability between the users’ behavior states. The frequency of screen states (on or off) over a 24-hour period was used to calculate screen entropy. For calculation details, refer to [44,54]	Higher screen entropy reflects a more distributed unlock of the screen (ie, high variability). Lower values indicate that users screen is more often in one state (ie, low variability).
	Screen normalized entropy	Normalized entropy is the entropy divided by the logarithm of the number of states (N)	Entropy divided by log(N)
**Call**			
	Missed calls count	Number of missed calls	Count
	Missed calls distinct contacts	Number of distinct contacts whose calls were missed	Count
	Incoming calls count	Number of incoming calls	Count
	Incoming calls distinct contacts	Number of distinct contacts whose calls were answered	Count
	Incoming calls duration	Duration of incoming calls	Time in hours
	Incoming calls entropy	Entropy is a measurement of the degree of variability between the users’ behavior states. The duration of incoming calls over a 24-hour period was used to calculate incoming calls entropy. For calculation details, refer to [44,54]	Higher incoming calls entropy reflects a more distributed call duration across incoming calls (ie, high variability)
	Outgoing calls count	Number of outgoing calls	Count
	Outgoing calls distinct contacts	Number of distinct contacts who were called.	Count
	Outgoing calls duration	Duration of outgoing calls	Time in hours
	Outgoing calls entropy	Entropy is a measurement of the degree of variability between the users’ behavior states. The duration of outgoing calls over a 24-hour period was used to calculate outgoing calls entropy. For calculation details, refer to [44,54]	Higher outgoing calls entropy reflects a more distributed call duration across outgoing calls (ie, high variability)
**Location**			
	Total distance	Total distance (haversine) between tracked location coordinates	Distance in km
	Location variance	Logarithm of the combined variance in latitude and longitude [44,48-50]	
	Moving to static ratio	Ratio of moving states (speed >1 km/h) to static states (speed <1 km/h) [50]	Ratio; higher values reflect more moving states compared to static states
	Number of significant clusters	Clusters are determined by k-means clustering of stationary location coordinates (speed <1 km/h). Clusters needed to be 400 meters from each other. Pauses within 200 meters of cluster were counted as cluster visits after the initial clustering. Only clusters with a time duration of 10 minutes were counted as significant. For further details, see [44,48-51]	Count
	Staying time at clusters	Time spent at significant clusters	Time in hours
	Time at top 1 location	Total time spent at the most significant cluster	Time in hours
	Time at top 2 location	Total time spent at the second most significant cluster	Time in hours
	Time at top 3 location	Total time spent at the third most significant cluster	Time in hours
	Location entropy	Entropy is a measurement of the degree of variability between the users’ behavior states. The duration at significant clusters over a 24-hour period was used to calculate location entropy. For calculation details, refer to [44,48-50,54]	Higher location entropy reflects more distributed time spent at significant clusters. Lower values indicate that users spent more time at some significant clusters.
	Location normalized entropy	Entropy divided by the logarithm of the number of significant clusters (N)	Entropy divided by the log(N)
	Location circadian movement	The extent to which a person’s visits at significant clusters follow a 24-hour circadian rhythm. For further details, see [44,50,52,53,56]	Low values indicate a break from routine, whereas high values indicate that a person followed a daily routine.
	Time spent at nonsignificant clusters (at outliers)	Time spent at nonsignificant clusters divided by the time spent at all significant cluster	Ratio; higher values indicate more time spent at nonsignificant clusters

### Preprocessing and Missing Data Handling

To account for missing data in the dataset, we performed multiple imputations by chained equations [42]. For a missingness overview before imputation, see Multimedia Appendix 5. As outlined before, we constructed 14-day periods consisting of the PHQ-8 and sensing features per day. The following imputations were conducted on the day level (ie, 14 days for each participant and episode). Given the nested data structure (ie, multiple days of the same participant), we applied 2-level predictive mean matching with random intercepts for all variables. A total of 20 complete datasets were obtained. Convergence was achieved after 10 iterations. In a second step, we aggregated the data from the daily level in each imputed dataset to the episode level to match the data structure of the PHQ-8 and sensing features. Aggregation consisted of the mean and SD across the 14 days. Therefore, each imputed dataset contained a single data entry for each person consisting of the average depression severity in the last 14 days (PHQ-8) and the average and SD of the sensing features in the last 14 days.

### Statistical Analysis

Given the exploratory character of this study, we decided not to report any *P* values. Instead, all analyses report on the point estimates and corresponding 95% CIs. Throughout the analyses, we conducted analyses on each imputed dataset separately and pooled the data using Rubin’s rule with Barnhard-Rubin adjustment for degrees of freedom [42,57-59]. The analysis strategy was structured in 3 steps. First, we calculated bivariate between-person Pearson correlations (*r*) and their 95% CIs between EMA and sensing features with depression. The correlation is a statistical measure of the magnitude of a linear relationship between 2 variables. It ranges from –1 (perfect negative linear relationship) to +1 (perfect positive linear relationship). Since higher values of the PHQ-8 indicate higher levels of depression, positive correlations between a feature and the PHQ-8 imply that higher values of this feature are associated with higher depression. However, correlations do not allow any causal inference. See Table 1 for interpretation guidance on all features.

Second, variables with non-zero correlation CIs were included as candidates in cluster-wise regression analyses (ie, limited to EMA, screen, app, location, and call features only). The best models per cluster were determined by stepwise backward exclusion of predictors with zero-including CIs. Predictors, starting with the least influential (standardized β) and broadest zero-including CI, were removed one at a time, and regression models were refitted and compared against each other based on adjusted *R*^2^ after each step. This stepwise backward elimination process was continued until all predictors with zero-including CIs were excluded and regression models no longer improved regarding adjusted *R*^2^. For each final cluster-wise regression, we determined the adjusted *R*^2^ and its 95% CI to quantify the explained variance in depression severity.

In the third step, we constructed regression models (1) combining the different sensing feature clusters in one model to evaluate the potential of smart sensing as a stand-alone paradigm and (2) including EMA and sensing feature clusters to evaluate their combined potential. As before, predictors were eliminated following stepwise backward exclusion. Overall model performance was evaluated based on adjusted *R*^2^. Differences in adjusted *R*^2^ and the information criteria Akaike information criterion and Bayesian information criterion were used for model comparisons. Model parameters were standardized and adjusted for age and gender in sensitivity analysis.

### Software

All analyses and data preparation were conducted in R. The mice and miceadds packages were used for the imputation and pooling of analysis results [42,60]. For a full list of all packages, see Multimedia Appendix 6.

## Results

### Overview

A total of 201 participants answered at least 1 PHQ-8. In total, 94 of the responders were excluded due to poor data quality (>50% average missingness in EMA and sensing features). Hence, a total of 107 participants were included in the analysis.

The mean age of the participants was 22.81 (SD 7.32), with the oldest participant being 56 years and the youngest 18 years. In total, 83 (77.6%) of the participants identified themselves as female (male: n=24, 22.4%). On average, participants showed subclinical depression levels (mean 5.82, SD 4.44), with 20 (18.7%) above the PHQ-8 ≥10 cutoff indicating clinically relevant depression severity. For a summary of the PHQ-8 item level, see Multimedia Appendix 7.

EMA features revealed an average valence of 65.98 (SD 12.82), arousal of 50.17 (SD 12.16), and stress of 38.85 (SD 15.66). Daily screen usage of the smartphone was 2.72 hours (SD 2.82) on average. A complete summary of all k=102 features and their respective means and SDs can be found in Multimedia Appendix 8.

### Correlations

Correlation analyses revealed small to medium correlations of EMA and sensing features with depression severity. Average valence ratings showed the highest correlation to depression severity of the EMA features (*r*=–0.55, 95% CI –0.67 to –0.41), while the SD of app frequency entropy in app usage (*r*=–0.19, 95% CI –0.37 to –0.00), mean duration of outgoing calls (*r*=0.25, 95% CI 0.04 to 0.43), location routine index (*r*=0.23, 95% CI 0.02 to 0.42), and the average screen duration (*r*=0.37, 95% CI 0.20 to 0.53) were the strongest features in their respective clusters. See Table 2 for a summary of all correlations with zero, excluding CIs. The complete correlation summary of all investigated features can be found in Multimedia Appendix 9.

**Table 2:** Pooled bivariate correlation results between depression and features.^a^

				*r* (95% CI)
**Ecological momentary assessment features**				
	Average valence		–0.55 (–0.67 to –0.41)	
	Average satisfaction with social quality		–0.51 (–0.64 to –0.35)	
	Average sleep quality		–0.50 (–0.63 to –0.34)	
	Average arousal		–0.42 (–0.57 to –0.25)	
	Average social quantity		–0.39 (–0.54 to –0.22)	
	Average nutrition		–0.25 (–0.45 to –0.03)	
	SD of valence		0.23 (0.04 to 0.41)	
	Average stress		0.42 (0.25 to 0.56)	
**App features**				
	SD of frequency entropy		–0.19 (–0.37 to –0.00)	
**Call features**				
	Average incoming call duration		0.21 (0.00 to 0.39)	
	SD of incoming call duration		0.21 (0.01 to 0.40)	
	Average outgoing call duration		0.25 (0.04 to 0.43)	
	SD of outgoing call duration		0.25 (0.04 to 0.44)	
**Features**				
		Average location routine index	0.23 (0.02 to 0.42)	
**Screen features**				
		Average total screen duration	0.23 (0.04 to 0.40)	
		Average max screen duration	0.24 (0.05 to 0.41)	
		Average SD of screen episode count	0.27 (0.09 to 0.44)	
		Average duration of screen episodes	0.37 (0.20 to 0.53)	

^a^Correlations are pooled correlations based on multiple imputations. Only correlations with their 95% CI excluding zero are displayed. A full correlation summary can be found in Multimedia Appendix 9. For feature definition and interpretation, refer to Table 1.

### Regression

We included the features identified in the correlation analysis in step-wise regression analyses to investigate their incremental contribution to the explained variance in depression severity. The final regression model using EMA features included average valence (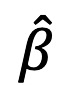
=–0.39, 95% CI –0.58 to –0.21) and social quality as predictors (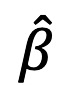
=–0.29, 95% CI –0.48 to –0.10). Combined, they explained 35.28% of the variance (95% CI 20.73% to 49.64%).

In the app cluster, SD of app frequency entropy (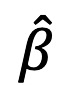
=–0.19, 95% CI –0.38 to –0.00) explained adjusted *R*^2^=2.81% of the variance (95% CI 0.00%-12.02%), while location routine index (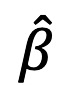
=0.23, 95% CI 0.02 to 0.44) explained adjusted *R*^2^=4.39% (95% CI 0.00% to 16.71%) in the location cluster. From the call cluster, the SD of incoming call duration (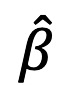
=0.20, 95% CI 0.03 to 0.41) and average outgoing call duration (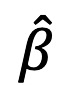
=0.24, 95% CI 0.04 to 0.44) were the final included predictors explaining adjusted *R*^2^=8.68% of the variance (95% CI 0.88% to 22.32%). Of all 4 candidates in the screen cluster, the average screen duration was the only included predictor in the final model (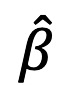
=0.37, 95% CI 0.19 to 0.55; adjusted *R*^2^=13.09%, 95% CI 3.40% to 26.65%).

Combining all sensing features in a parsimonious model yielded a model explaining adjusted *R*^2^=20.45% of the variance (95% CI 7.81% to 35.59%) with average screen duration (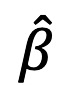
=0.39, 95% CI 0.21 to 0.56), SD of app frequency entropy (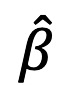
=–0.19, 95% CI –0.36 to –0.02), and SD of incoming call duration (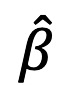
=0.21, 95% CI 0.02 to 0.41) as predictors.

The highest variance was explained when combining EMA and sensing features: adj. *R*^2^=45.15% of the variance (95% CI 30.39% to 58.53%). Features included in this prediction model were average valence (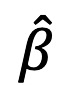
=–0.36, 95% CI –0.53 to –0.19), social quality (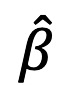
=–0.24, 95% CI –0.41 to –0.06), average screen duration (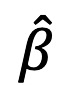
=0.22, 95% CI 0.07 to 0.37), SD of app frequency entropy (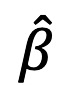
= –0.17, 95% CI –0.31 to –0.02), and average duration of outgoing calls (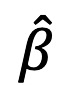
=0.17, 95% CI 0.01 to 0.33). See Table 3 for a summary of the parsimonious EMA, sensing, and combined regression models.

**Table 3:** Regression results for depression predicted by ecological momentary assessment and smartphone features in stand-alone and combined models.^a^

			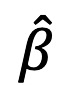 (95% CI)		Adjusted *R*^2^ (95% CI), %		AIC^b^		BIC^c^		Δ adjusted *R*^2^, %
**EMA^d^ cluster**					35.28 (20.73 to 49.64)		262.45		273.14		N/A^e^
	Average valence	–0.39 (–0.58 to –0.21)									
	Average social quality	–0.29 (–0.48 to –0.10)									
**Sensing cluster**					20.45 (7.81 to 35.59)		284.53		297.90		EMA: –14.83
	Average screen duration	0.39 (0.21 to 0.56)									
	Average app frequency entropy	–0.19 (–0.36 to –0.02)									
	Incoming call SD	0.21 (0.02 to 0.41)									
**Combined**					45.15 (30.39 to 58.53)		249.42		268.13		EMA: 9.87; sensing: 24.70
	Average valence	–0.36 (–0.53 to –0.19)									
	Average social quality	–0.24 (–0.41 to –0.06)									
	Average screen duration	0.22 (0.07 to 0.37)									
	Average app frequency entropy	–0.17 (–0.31 to –0.02)									
	Average duration of outgoing calls	0.17 (0.01 to 0.33)									

^a^All results were obtained by pooling results from multiple imputations according to Rubin’s rule. All estimates were fully standardized. For feature definition and interpretation, refer to Table 1.

^b^AIC: Akaike information criterion.

^c^BIC: Bayesian information criterion.

^d^EMA: ecological momentary assessment.

^e^Not applicable.

Results were robust when adjusting for age and gender, yielding nonsignificant main effects in the parsimonious EMA (age: –0.08 to 0.23; gender: –0.06 to 0.26), sensing (age: –0.16 to 0.21; gender: –0.26 to 0.10), and combined EMA and sensing model (age: –0.09 to 0.22; gender: –0.09 to 0.21).

## Discussion

### Principal Findings

To explore the potential of smart sensing for depression, this study investigated the bivariate correlations and explainable variance in regression models built on smartphone sensors and EMA features. Across sensor modalities, we found small correlations between smart sensing features and depression severity. Combined smart sensing features could explain 20.45% (95% CI 7.81%-35.59%) of the depression severity variance in a parsimonious model. Conversely, we found small to medium correlations for EMA features, which could explain 35.28% (95% CI 20.73%-49.64%) of the variance. The best model was the combination of smart sensing and EMA features, which explained 45.15% (95% CI 30.39%-58.53%; Δ adjusted *R*^2^ to smart sensing only: 24.70%; Δ adjusted *R*^2^ to EMA only: 9.87%).

### Comparison to Prior Work

The EMA findings are in line with previous studies and reviews highlighting the potential of EMA as a continuous assessment to infer depression severity [35,37,40,61,62]. However, while the bivariate correlations, as well as the explained variance, are higher for EMA compared to features obtained from smartphone sensors, it is important to note that the sensing cluster alone could explain about 20% of the variance. Given the unobtrusive nature of the collection of sensor data, this approach has a crucial advantage over EMA, which requires active user or patient involvement over a long period (eg, multiple daily responses over 14 days). In particular, for clinical application, it should be evaluated whether the additional burden for patients to answer EMA is proportional to the gain in explained variance. Furthermore, various issues in EMA (eg, interpretation of momentary questions, usage of comparison standards) could be avoided by the collection of objective sensor data [19,21,62,63]. That said, to maximize the explained variance, the combination of sensor features and EMA seems to be best. This result extends the findings by Moshe et al [35], who previously evaluated GPS features in conjunction with physiological wearable data and EMA similarly, showing that the combination of sensing features with EMA yields the best regression model for depression severity.

Although these findings seem promising, it is also important to note that this study and others so far are exploratory [34-36,64]. Although we have more than 2 decades of research on EMA and its application for mental health [18,37], the field of smart sensing is still in its infancy. Facing heterogenous methodology and study quality, as well as potential publication bias in the field, confirmatory studies are highly needed in the field of smart sensing before clinical application [34,36,64,65]. Besides, it is important to note that this study followed a rather data-driven approach to investigating features, which were collectible by the here-used framework. In the context of smart sensing, a central question is which features are needed and provide an incremental benefit. Although this study can give first insights into this topic, predictors like screen duration, app usage entropy, and call features (eg, SD in incoming calls) should only be incorporated in clinical systems if replicated in future studies. Also, an extension to other sensors (eg, language analysis based on LIWC or sentiment analysis) [66-70], app content (eg, usage of social media apps) [71], network usage [28], and the combination with other wearable devices (eg, biophysiological data from smart watches) or different data sources (eg, journaling data) would be a promising addition [23,24,35,67].

However, a closer inspection is needed not only for smart sensing features but also for EMA. For example, our study highlighted that many of the investigated features (eg, arousal or physical activity) did not hold any incremental benefit besides valence and social quality. Since EMA is associated with additional burden for patients, it is especially important to identify the core set of items that maximize predictive power while reducing the item load. Despite a long research history, surprisingly little systematic and meta-analytical evidence is available on which questions should be asked in the context of depression and more precisely when (eg, morning or evening) and on which schedule (eg, multiple fixed time points or microrandomized assessments) [37,63,72-74].

Besides the empirical evidence for the applicability of smart sensing, questions of acceptance, data security and privacy, and ethical challenges surrounding smart sensing need to be addressed [19,75-77]. For instance, recent studies found only moderate acceptance of smart sensing in the context of mental health and highlighted the impact of data types and recipients in smart sensing [77-79]. Only when these barriers and challenges are overcome, can smart sensing unfold its potential fully.

Lastly, we want to point out that the current developments in smart sensing for mental health mainly focus on the prediction of psychopathology [24,26,27,36,64]. Future studies could further advance the field by not only focusing on pathology but also investigating applications to assess risk factors (eg, loneliness, stress) or mediators and mechanisms of change (eg, rumination, therapeutic alliance). Understanding the underlying process of how treatment works is a crucial step to optimizing treatments and pathways in mental health care [80-83]. Since smart sensing allows for a fine-grained and unobtrusive assessment, it might become a promising and feasible paradigm to unveil the mechanism of change in mental health care and better understand the dynamics of therapeutic processes.

### Limitations

In addition to the already highlighted exploratory nature of this study, we would like to emphasize a few limitations of this study. First, this study followed a cross-sectional design investigating which predictors explain the variance between persons. Hence, any causal interpretation of the results is not eligible and would require different study designs. For instance, the increased smartphone usage (eg, screen duration) could be caused by depression, but also vice versa or even explained by a third variable. Also, the analysis of trajectories and dynamics over time was not in the scope of this study. Based on the multiple episodes of participants in this study, a longitudinal perspective would be a valuable addition to this study and a field with so far underrepresented research on longitudinal models [34,84,85].

Second, the present sample was a convenient sample recruited in the general population, which resulted in a rather young (mean 22.81, SD 7.32, range 18-56) and unequal gender distribution (female: 77.6%). Furthermore, only 18.7% of the participants showed clinically relevant symptomology. Therefore, the generalizability of the findings to other samples, especially clinical samples, remains open. However, by showing the feasibility of smart sensing predictions and their incremental benefit in explaining variance in depression, this study lays a strong foundation to move to clinical populations and studies. To further increase the quality of clinical studies, also methodological points need to be addressed; for instance, self-report instruments, as applied in this study (ie, PHQ-8), are prone to several sources of bias (eg, social desirability or recall biases). Hence, the application of more reliable and valid assessments like clinician ratings or medical diagnosis should be considered in future studies alongside measurements, which maximize reliability in a specific depression severity range of interest (eg, high reliability in subclinical or severe depression levels) [86-88].

Third, alongside the sample characteristics, the sample size needs to be considered. Adequate sample size is key to designing confirmatory studies aiming to test an assumed clinically relevant effect with sufficient power. In depression research, a standardized mean difference of 0.24, which transfers to a correlation of *r*=0.12 [89], is argued to be a clinically relevant effect [90]. However, to be able to test such a correlation with sufficient power (eg, 80%), a sample size of N=542 would be required (assuming a 2-sided test with α=5%, following a bivariate normal model). Hence, our study was highly underpowered to test for a minimally clinically relevant correlation of *r*=0.12. Accordingly, we did not report any *P* values and solely reported on the 95% CIs to provide a range of reasonable assumable magnitudes of estimates, which could guide future studies in their sample size planning.

Lastly, we would like to emphasize that we opted for linear regression analysis to investigate the incremental contribution of various predictors and allow for direct comparison of various models. This method provides a straightforward approach to answering the present research question. However, sensor data are also very complex, and previous studies have highlighted the potential of nonlinear machine learning models for depression severity to fully exploit the smart sensing data [25,28]. Although previously applied to classification, machine learning models like extreme gradient-boosted regression trees may also be promising in regression. On the flip side, these models go hand in hand with challenges such as overfitting and difficulties in the interpretation and explanations of the models [25,28,91]. Therefore, even if proven to improve the predictive accuracy, it should be carefully considered whether the complexity and downsides (eg, explainability) of potential machine learning models justify their usage over simpler but easy-to-interpret statistical regression models [92].

### Conclusions

Smart sensing and EMA provide potent paradigms to infer and predict depression severity. Our results show that EMA and sensing features alone can substantially explain variance in depression severity. In isolation, EMA was superior to sensing features in terms of explained variance. However, sensing features alone could explain about 20% of the variance, emphasizing the potential of this unobtrusive and objective assessment of depression. To maximize explainable variance, EMA and sensing features should be combined. However, while these findings are promising, confirmatory studies, particularly in clinical settings and samples, are highly needed before robust conclusions can be drawn.

## Appendix

Multimedia Appendix 1 STROBE checklist.

Multimedia Appendix 2 Sensitivity analysis on study exclusion.

Multimedia Appendix 3 Patient Health Questionnaire items.

Multimedia Appendix 4 Ecological momentary assessment items.

Multimedia Appendix 5 Overview of missingness per feature before multiple imputation.

Multimedia Appendix 6 Software information.

Multimedia Appendix 7 Additional sample characteristics.

Multimedia Appendix 8 Feature means and SDs.

Multimedia Appendix 9 Full correlations between depression and features.

## Abbreviations

EMA: ecological momentary assessment
MDD: major depressive disorder
PHQ-8: 8-item Patient Health Questionnaire
STROBE: Strengthening the Reporting of Observational Studies in Epidemiology

## Data Availability

The primary data obtained in this observation study can be provided by HB and YT on reasonable request. Data-sharing agreements may have to be signed depending on the request. Requests should be directed at the corresponding author (YT). Support is depending on available resources.
