# Supplementary material for: Investigating Smartphone-Based Sensing Features for Depression Severity Prediction: Observation Study
Source: J Med Internet Res. 2025 Jan 30;27:e55308. doi: 10.2196/55308 (PMC11826944; doi:10.2196/55308)
Supplement: Multimedia Appendix 2 [file jmir_v27i1e55308_app2.docx]

## Multimedia Appendix 2: Sensitivity Analysis on Study Exclusion.

Due to technical issues with the sensing framework, several participants needed to be excluded. We assume that technical issues were occurring randomly and had no biasing influence on the results. Sensitivity analysis on age, gender, and depression supported this assumption.

| **Variable** | **M Included** | **M Excluded** | **SD Included** | **SD Excluded** | **Adj. P** |
| --- | --- | --- | --- | --- | --- |
| age | 22.81 | 22.95 | 7.32 | 9.80 | .975 |
| Gender (female) | 83 | 74 | 0.42 | 0.35 | .370 |
| Interest  (PHQ-8 Item 1) | 0.82 | 0.72 | 0.75 | 0.66 | .582 |
| Depression (PHQ-8 Item 2) | 0.67 | 0.73 | 0.79 | 0.81 | .854 |
| Sleep  (PHQ-8 Item 3) | 0.93 | 1.26 | 0.91 | 0.95 | .127 |
| Energy  (PHQ-8 Item 4) | 1.24 | 1.24 | 0.81 | 0.84 | .975 |
| Appetite  (PHQ-8 Item 5) | 0.64 | 0.76 | 0.76 | 0.91 | .582 |
| Self-worth (PHQ-8 Item 6) | 0.56 | 0.67 | 0.83 | 0.89 | .582 |
| Concentration (PHQ-8 Item 7) | 0.75 | 0.72 | 0.77 | 0.77 | .960 |
| Agitation  (PHQ-8 Item 8 | 0.14 | 0.27 | 0.44 | 0.56 | .370 |

Note: a) Included and excluded participants were compared using t-tests for age and PHQ-8 variables, and a chi-square test for gender frequency. P-values are Bonferroni-Holm adjusted.
