# Supplementary material for: Investigating Smartphone-Based Sensing Features for Depression Severity Prediction: Observation Study
Source: J Med Internet Res. 2025 Jan 30;27:e55308. doi: 10.2196/55308 (PMC11826944; doi:10.2196/55308)
Supplement: Multimedia Appendix 3 [file jmir_v27i1e55308_app3.docx]

## Multimedia Appendix 3: PHQ-8 Items

The PHQ-8 assess the to which extend participants were bothered by symptoms over the last 2 weeks: “Over the last 2 weeks, how often have you been bothered by any of the following problems?”

For each symptom participants can answer with 0 = Not at all, 1 = Several days, 2 = More than half the days, or 3 = nearly every day.

| Item number | Item |
| --- | --- |
| 1 | Little interest or pleasure in doing things |
| 2 | Feeling down, depressed, or hopeless |
| 3 | Trouble falling or staying asleep, or sleeping too much |
| 4 | Feeling tired or having little energy |
| 5 | Poor appetite or overeating |
| 6 | Feeling bad about yourself – or that you are a failure or have let yourself or your family down |
| 7 | Trouble concentrating on things, such as reading the newspaper or watching television |
| 8 | Moving or speaking so slowly that other people could have noticed. Or the opposite – being so fidgety restless that you have been moving around a lot more than usual |

Please see references [46,47] for more details on the PHQ-8.
