# Supplementary material for: Investigating Smartphone-Based Sensing Features for Depression Severity Prediction: Observation Study
Source: J Med Internet Res. 2025 Jan 30;27:e55308. doi: 10.2196/55308 (PMC11826944; doi:10.2196/55308)
Supplement: Multimedia Appendix 4 [file jmir_v27i1e55308_app4.docx]

## Multimedia Appendix 4: EMA Items

| **German** | **English** |
| --- | --- |
| Wie gut hast du geschlafen?  (sehr schlecht 0 – sehr gut 100) | How did you sleep?  (very poorly 0 - very good 100) |
| Wie geht es dir?  (sehr schlecht 0 – sehr gut 100) | How do you feel?  (very poorly 0 - very good 100) |
| Wie aktiv fühlst du dich?  (sehr träge 0 – überaktiviert 100) | How active are you?  (very low 0 - very high 100) |
| Wie gestresst fühlst du dich?  (extrem entspannt 0 – sehr gestresst 100) | How stressed are you?  (very relaxed 0 - very stressed 100) |
| Wie gesund war deine Mahlzeit?  (sehr ungesund 0 – sehr gesund 100) | How healthy was your meal?  (very unhealthy 0 - very healthy 100) |
| Wie zufrieden bist du mit der Beziehung zu deinen Freunden und deiner Familie?  (sehr unzufrieden 0 – vollkommen zufrieden 100) | How satisfied are you with the quality of your relationships with friends and family?  (very unsatisfied 0 - very satisfied 100) |
| Wie zufrieden bist du mit der Häufigkeit deiner sozialen Kontakte?  (sehr unzufrieden 0 – vollkommen zufrieden 100) | How satisfied are you with the frequency of your social contacts?  (very unsatisfied 0 - very satisfied 100) |
| Wie zufrieden bist du mit deiner körperlichen Aktivität?  (gar nicht anstrengend 0 – sehr anstrengend 100) | How satisfied are you with your physical activity?  (very unsatisfied 0 - very satisfied 100) |
