# Supplementary material for: Investigating Smartphone-Based Sensing Features for Depression Severity Prediction: Observation Study
Source: J Med Internet Res. 2025 Jan 30;27:e55308. doi: 10.2196/55308 (PMC11826944; doi:10.2196/55308)
Supplement: Multimedia Appendix 5 [file jmir_v27i1e55308_app5.docx]

## Multimedia Appendix 5: Overview of Missingness per Feature Before Multiple Imputation

Please refer to table 1 in the methods section for the definition and interpretation of the included features. Features were calculated on daily level and in a second step aggregated across the 14-day period for each participant. Missingness refers to the average amount of missing data pooled across persons and 14-day periods.

| Feature | Percent Missing |
| --- | --- |
| phq_sum | 0.00% |
| Valence_mean | 7.91% |
| Arousal_mean | 7.98% |
| Stress_mean | 8.10% |
| Sleep_quality_mean | 6.67% |
| Social_Inclusion_quantitiy_mean | 8.16% |
| Social_Inclusion_quality_mean | 8.16% |
| nutrition_mean | 53.52% |
| sport_mean | 64.80% |
| apps_day_frequencyentropyall | 1.87% |
| apps_day_countall | 1.87% |
| apps_day_meandurationall | 1.87% |
| apps_day_ridurationall | 1.87% |
| missed_count | 34.52% |
| missed_distinctcontacts | 34.52% |
| incoming_count | 34.52% |
| incoming_distinctcontacts | 34.52% |
| incoming_meanduration | 34.52% |
| incoming_sumduration | 34.52% |
| incoming_maxduration | 34.52% |
| incoming_entropyduration | 34.52% |
| outgoing_count | 34.52% |
| outgoing_distinctcontacts | 34.52% |
| outgoing_meanduration | 34.52% |
| outgoing_sumduration | 34.52% |
| outgoing_maxduration | 34.52% |
| outgoing_entropyduration | 34.52% |
| stdlengthstayatclusters | 40.75% |
| circadianmovement | 41.87% |
| loglocationvariance | 41.87% |
| movingtostaticratio | 40.75% |
| locationentropy | 44.24% |
| locationroutineindex | 44.30% |
| totaldistance | 39.94% |
| normalizedlocationentropy | 44.24% |
| numberlocationtransitions | 40.75% |
| locationvariance | 41.62% |
| meanlengthstayatclusters | 40.75% |
| outlierstimepercent | 40.75% |
| maxlengthstayatclusters | 40.75% |
| numberofsignificantplaces | 40.75% |
| timeattop1 | 44.24% |
| timeattop2 | 61.56% |
| timeattop3 | 68.97% |
| screen_countepisode | 1.62% |
| screen_sumduration | 1.62% |
| screen_maxduration | 1.62% |
| screen_avgduration | 1.62% |
| screen_ri_alldays_avg | 1.62% |
| screen_ri_alldays_range | 1.62% |
| screen_entropy | 1.62% |
| screen_normalisedEntropy | 1.62% |
| age | 0.00% |
| gender | 0.00% |
