# Supplementary material for: Investigating Smartphone-Based Sensing Features for Depression Severity Prediction: Observation Study
Source: J Med Internet Res. 2025 Jan 30;27:e55308. doi: 10.2196/55308 (PMC11826944; doi:10.2196/55308)
Supplement: Multimedia Appendix 6 [file jmir_v27i1e55308_app6.docx]

## Multimedia Appendix 6: Software information

R version 4.3.2 (2023-10-31)

attached base packages:

[1] stats graphics grDevices utils datasets methods base

other attached packages:

[1] corrplot_0.92 texreg_1.39.3 lavaan_0.6-17 mitml_0.4-5 lmerTest_3.1-3 lme4_1.1-35.1

[7] Matrix_1.6-1.1 psych_2.3.12 ggmice_0.1.0 ggplot2_3.5.1 lubridate_1.9.3 multiUS_1.2.3

[13] here_1.0.1 miceadds_3.16-18 tidyr_1.3.1 readxl_1.4.3 magrittr_2.0.3 dplyr_1.1.4

[19] readr_2.1.4 haven_2.5.4 mice_3.16.0

loaded via a namespace (and not attached):

[1] tidyselect_1.2.1 fastmap_1.1.1 digest_0.6.33 rpart_4.1.21 timechange_0.2.0

[6] lifecycle_1.0.4 survival_3.5-7 compiler_4.3.2 rlang_1.1.3 tools_4.3.2

[11] utf8_1.2.4 yaml_2.3.8 knitr_1.45 ggsignif_0.6.4 bit_4.0.5

[16] mnormt_2.1.1 abind_1.4-5 numDeriv_2016.8-1.1 withr_3.0.0 purrr_1.0.2

[21] stats4_4.3.2 nnet_7.3-19 grid_4.3.2 fansi_1.0.6 ggpubr_0.6.0

[26] jomo_2.7-6 colorspace_2.1-0 scales_1.3.0 iterators_1.0.14 MASS_7.3-60

[31] cli_3.6.2 rmarkdown_2.27 crayon_1.5.2 generics_0.1.3 rstudioapi_0.16.0

[36] httr_1.4.7 tzdb_0.4.0 minqa_1.2.6 DBI_1.2.0 splines_4.3.2

[41] parallel_4.3.2 cellranger_1.1.0 mitools_2.4 vctrs_0.6.5 boot_1.3-28.1

[46] glmnet_4.1-8 carData_3.0-5 car_3.1-2 hms_1.1.3 bit64_4.0.5

[51] rstatix_0.7.2 foreach_1.5.2 glue_1.7.0 nloptr_2.0.3 pan_1.9

[56] codetools_0.2-19 shape_1.4.6 gtable_0.3.5 quadprog_1.5-8 munsell_0.5.1

[61] tibble_3.2.1 pillar_1.9.0 htmltools_0.5.7 R6_2.5.1 rprojroot_2.0.4

[66] pbivnorm_0.6.0 vroom_1.6.5 evaluate_0.23 lattice_0.21-9 backports_1.4.1

[71] broom_1.0.5 Rcpp_1.0.12 nlme_3.1-163 xfun_0.42 forcats_1.0.0

[76] pkgconfig_2.0.3
