# Supplementary material for: Investigating Smartphone-Based Sensing Features for Depression Severity Prediction: Observation Study
Source: J Med Internet Res. 2025 Jan 30;27:e55308. doi: 10.2196/55308 (PMC11826944; doi:10.2196/55308)
Supplement: Multimedia Appendix 7 [file jmir_v27i1e55308_app7.docx]

## Multimedia Appendix 7: Additional Sample Characteristics

| **PHQ-8 Item** | **M** | **SD** |
| --- | --- | --- |
| Interest (PHQ-8 Item 1) | 0.82 | 0.75 |
| Depression (PHQ-8 Item 2) | 0.67 | 0.79 |
| Sleep (PHQ-8 Item 3) | 0.93 | 0.91 |
| Energy (PHQ-8 Item 4) | 1.24 | 0.81 |
| Appetite (PHQ-8 Item 5) | 0.64 | 0.76 |
| Self-worth (PHQ-8 Item 6) | 0.56 | 0.83 |
| Concentration (PHQ-8 Item 7) | 0.75 | 0.77 |
| Agitation (PHQ-8 Item 8 | 0.14 | 0.44 |
