# Supplementary material for: Investigating Smartphone-Based Sensing Features for Depression Severity Prediction: Observation Study
Source: J Med Internet Res. 2025 Jan 30;27:e55308. doi: 10.2196/55308 (PMC11826944; doi:10.2196/55308)
Supplement: Multimedia Appendix 8 [file jmir_v27i1e55308_app8.docx]

## Multimedia Appendix 8: Features Means and Standard Deviations

Please refer to Table 1 for the definition of features and their interpretation (e.g., units). The sample characteristics below refer to the aggregated values: Features were first aggregated across the 14-day periods per person, and then across participants. For instance, for the feature valence each participant provided 14 daily values, which were aggregated to a mean value and standard deviation (= per person mean and standard deviation). In a second step, these per-person means and standard deviations where descriptively analyzed (see sample means and standard deviations below).

| **Feature** | **M** | **SD** |
| --- | --- | --- |
| Valence_mean_ema_avg | 65.98 | 12.82 |
| Valence_mean_ema_std | 11.60 | 4.95 |
| Arousal_mean_ema_avg | 50.17 | 12.16 |
| Arousal_mean_ema_std | 13.64 | 5.46 |
| Stress_mean_ema_avg | 38.85 | 15.66 |
| Stress_mean_ema_std | 12.99 | 5.52 |
| Sleep_quality_mean_ema_avg | 64.01 | 14.20 |
| Sleep_quality_mean_ema_std | 16.13 | 6.62 |
| Social_Inclusion_quantitiy_mean_ema_avg | 61.93 | 23.48 |
| Social_Inclusion_quantitiy_mean_ema_std | 17.72 | 13.30 |
| Social_Inclusion_quality_mean_ema_avg | 73.00 | 18.48 |
| Social_Inclusion_quality_mean_ema_std | 8.97 | 5.64 |
| nutrition_mean_ema_avg | 58.12 | 11.52 |
| nutrition_mean_ema_std | 13.94 | 4.17 |
| sport_mean_ema_avg | 21.80 | 16.94 |
| sport_mean_ema_std | 22.29 | 14.55 |
| apps_frequencyentropyall_app_avg | 1.83 | 0.33 |
| apps_frequencyentropyall_app_std | 0.27 | 0.11 |
| apps_countall_app_avg | 123.25 | 73.93 |
| apps_countall_app_std | 45.58 | 27.59 |
| apps_meandurationall_app_avg | 1.53 | 1.51 |
| apps_meandurationall_app_std | 0.79 | 1.26 |
| apps_ridurationall_app_avg | 0.04 | 0.02 |
| apps_ridurationall_app_std | 0.01 | 0.01 |
| screen_countepisode_screen_avg | 89.79 | 52.75 |
| screen_countepisode_screen_std | 31.77 | 21.20 |
| screen_sumduration_screen_avg | 168.85 | 101.16 |
| screen_sumduration_screen_std | 72.97 | 45.01 |
| screen_maxduration_screen_avg | 40.45 | 32.15 |
| screen_maxduration_screen_std | 27.97 | 29.29 |
| screen_avgduration_screen_avg | 2.72 | 2.82 |
| screen_avgduration_screen_std | 1.71 | 2.05 |
| screen_ri_alldays_avg_screen_avg | 0.04 | 0.02 |
| screen_ri_alldays_avg_screen_std | 0.01 | 0.01 |
| screen_ri_alldays_range_screen_avg | 0.08 | 0.02 |
| screen_ri_alldays_range_screen_std | 0.02 | 0.01 |
| screen_entropy_screen_avg | 2.12 | 0.56 |
| screen_entropy_screen_std | 0.37 | 0.15 |
| screen_normalisedEntropy_screen_avg | 0.79 | 0.14 |
| screen_normalisedEntropy_screen_std | 0.09 | 0.04 |
| missed_count_call_avg | 0.67 | 0.69 |
| missed_count_call_std | 0.87 | 0.78 |
| missed_distinctcontacts_call_avg | 0.47 | 0.34 |
| missed_distinctcontacts_call_std | 0.55 | 0.25 |
| incoming_count_call_avg | 0.82 | 0.55 |
| incoming_count_call_std | 0.92 | 0.53 |
| incoming_distinctcontacts_call_avg | 0.64 | 0.34 |
| incoming_distinctcontacts_call_std | 0.65 | 0.23 |
| incoming_meanduration_call_avg | 3.99 | 4.79 |
| incoming_meanduration_call_std | 6.90 | 7.33 |
| incoming_sumduration_call_avg | 6.16 | 7.32 |
| incoming_sumduration_call_std | 10.45 | 10.65 |
| incoming_maxduration_call_avg | 5.24 | 6.15 |
| incoming_maxduration_call_std | 8.81 | 8.64 |
| incoming_entropyduration_call_avg | 0.11 | 0.13 |
| incoming_entropyduration_call_std | 0.21 | 0.17 |
| outgoing_count_call_avg | 2.02 | 1.54 |
| outgoing_count_call_std | 1.91 | 1.57 |
| outgoing_distinctcontacts_call_avg | 1.28 | 0.63 |
| outgoing_distinctcontacts_call_std | 1.00 | 0.48 |
| outgoing_meanduration_call_avg | 3.30 | 3.94 |
| outgoing_meanduration_call_std | 5.49 | 6.26 |
| outgoing_sumduration_call_avg | 6.82 | 8.39 |
| outgoing_sumduration_call_std | 9.50 | 9.95 |
| outgoing_maxduration_call_avg | 5.52 | 6.89 |
| outgoing_maxduration_call_std | 7.83 | 8.00 |
| outgoing_entropyduration_call_avg | 0.24 | 0.21 |
| outgoing_entropyduration_call_std | 0.32 | 0.18 |
| stdlengthstayatclusters_location_avg | 38.13 | 41.50 |
| stdlengthstayatclusters_location_std | 32.36 | 31.15 |
| circadianmovement_location_avg | -2.20 | 0.21 |
| circadianmovement_location_std | 0.25 | 0.08 |
| loglocationvariance_location_avg | -4.50 | 1.51 |
| loglocationvariance_location_std | 1.94 | 0.69 |
| movingtostaticratio_location_avg | 0.72 | 0.17 |
| movingtostaticratio_location_std | 0.15 | 0.10 |
| locationentropy_location_avg | 0.45 | 0.30 |
| locationentropy_location_std | 0.38 | 0.17 |
| locationroutineindex_location_avg | 2.72 | 3.12 |
| locationroutineindex_location_std | 1.17 | 1.06 |
| totaldistance_location_avg | 34.01 | 38.14 |
| totaldistance_location_std | 45.50 | 51.87 |
| normalizedlocationentropy_location_avg | 0.30 | 0.17 |
| normalizedlocationentropy_location_std | 0.25 | 0.11 |
| numberlocationtransitions_location_avg | 10.53 | 10.82 |
| numberlocationtransitions_location_std | 8.37 | 6.75 |
| locationvariance_location_avg | 0.06 | 0.18 |
| locationvariance_location_std | 0.17 | 0.60 |
| meanlengthstayatclusters_location_avg | 1.02 | 1.37 |
| meanlengthstayatclusters_location_std | 1.33 | 1.59 |
| outlierstimepercent_location_avg | 0.22 | 0.26 |
| outlierstimepercent_location_std | 0.17 | 0.15 |
| maxlengthstayatclusters_location_avg | 2.73 | 2.84 |
| maxlengthstayatclusters_location_std | 1.70 | 1.34 |
| numberofsignificantplaces_location_avg | 3.67 | 3.07 |
| numberofsignificantplaces_location_std | 2.41 | 1.86 |
| timeattop1_location_avg | 4.12 | 3.77 |
| timeattop1_location_std | 2.24 | 1.42 |
| timeattop2_location_avg | 0.80 | 0.58 |
| timeattop2_location_std | 0.75 | 0.45 |
| timeattop3_location_avg | 0.35 | 0.23 |
| timeattop3_location_std | 0.38 | 0.21 |
