# Supplementary material for: Investigating Smartphone-Based Sensing Features for Depression Severity Prediction: Observation Study
Source: J Med Internet Res. 2025 Jan 30;27:e55308. doi: 10.2196/55308 (PMC11826944; doi:10.2196/55308)
Supplement: Multimedia Appendix 9 [file jmir_v27i1e55308_app9.docx]

## Multimedia Appendix 9: Full correlations between depression and features.

Please refer to Table 1 for the definition of features and their interpretation (e.g., units). Features were first aggregated across the 14-day periods per person, and then across participants. For instance, for the feature valence each participant provided 14 daily values, which were aggregated to a mean value and standard deviation (= per person mean and standard deviation). All correlations below refer to the correlations with the PHQ-8 sum scores.

### EMA

| Feature | r | rse | fisher_r | fisher_rse | fmi | lower95 | upper95 |
| --- | --- | --- | --- | --- | --- | --- | --- |
| Mood_avg | -0.55 | 0.07 | -0.62 | 0.10 | 0.00 | -0.67 | -0.41 |
| Mood_std | 0.23 | 0.09 | 0.24 | 0.10 | 0.04 | 0.04 | 0.41 |
| Arousal_avg | -0.42 | 0.08 | -0.45 | 0.10 | 0.02 | -0.57 | -0.25 |
| Arousal_std | 0.07 | 0.10 | 0.07 | 0.10 | 0.01 | -0.13 | 0.25 |
| Stress_avg | 0.42 | 0.08 | 0.45 | 0.10 | 0.00 | 0.25 | 0.56 |
| Stress_std | 0.16 | 0.10 | 0.16 | 0.10 | 0.03 | -0.03 | 0.34 |
| Sleep_quality_avg | -0.50 | 0.07 | -0.55 | 0.10 | 0.01 | -0.63 | -0.34 |
| Sleep_quality_std | 0.07 | 0.10 | 0.07 | 0.10 | 0.01 | -0.12 | 0.26 |
| Social_quantitiy_avg | -0.39 | 0.08 | -0.41 | 0.10 | 0.01 | -0.54 | -0.22 |
| Social_quantitiy_std | -0.07 | 0.10 | -0.07 | 0.10 | 0.02 | -0.26 | 0.13 |
| Social_quality_avg | -0.51 | 0.07 | -0.56 | 0.10 | 0.00 | -0.64 | -0.35 |
| Social_quality_std | 0.06 | 0.10 | 0.06 | 0.10 | 0.02 | -0.13 | 0.25 |
| nutrition_avg | -0.25 | 0.11 | -0.26 | 0.12 | 0.32 | -0.45 | -0.03 |
| nutrition_std | 0.11 | 0.12 | 0.11 | 0.12 | 0.34 | -0.13 | 0.33 |
| sport_avg | -0.01 | 0.14 | -0.01 | 0.14 | 0.49 | -0.27 | 0.25 |

### App

| Feature | r | rse | fisher_r | fisher_rse | fmi | lower95 | upper95 |
| --- | --- | --- | --- | --- | --- | --- | --- |
| frequencyentropyall_app_avg | 0.19 | 0.09 | 0.19 | 0.10 | 0.00 | 0.00 | 0.36 |
| frequencyentropyall_app_std | -0.19 | 0.09 | -0.20 | 0.10 | 0.00 | -0.37 | 0.00 |
| countall_app_avg | -0.01 | 0.10 | -0.01 | 0.10 | 0.00 | -0.20 | 0.18 |
| countall_app_std | -0.04 | 0.10 | -0.04 | 0.10 | 0.00 | -0.23 | 0.15 |
| meandurationall_app_avg | 0.08 | 0.10 | 0.08 | 0.10 | 0.01 | -0.12 | 0.26 |
| meandurationall_app_std | 0.05 | 0.10 | 0.05 | 0.10 | 0.02 | -0.15 | 0.23 |
| ridurationall_app_avg | 0.06 | 0.10 | 0.06 | 0.10 | 0.00 | -0.13 | 0.25 |
| ridurationall_app_std | 0.00 | 0.10 | 0.00 | 0.10 | 0.00 | -0.19 | 0.19 |

### Call

| feature | r | rse | fisher_r | fisher_rse | fmi | lower95 | upper95 |
| --- | --- | --- | --- | --- | --- | --- | --- |
| missed_count_avg | -0.16 | 0.10 | -0.16 | 0.10 | 0.05 | -0.35 | 0.03 |
| missed_count_std | -0.16 | 0.10 | -0.16 | 0.10 | 0.06 | -0.34 | 0.04 |
| missed_distinctcontacts_avg | -0.14 | 0.10 | -0.15 | 0.10 | 0.08 | -0.33 | 0.06 |
| missed_distinctcontacts_std | -0.16 | 0.10 | -0.16 | 0.10 | 0.09 | -0.35 | 0.04 |
| incoming_count_avg | -0.09 | 0.10 | -0.09 | 0.10 | 0.06 | -0.28 | 0.11 |
| incoming_count_std | -0.02 | 0.10 | -0.02 | 0.10 | 0.07 | -0.22 | 0.17 |
| incoming_distinctcontacts_avg | -0.07 | 0.10 | -0.07 | 0.11 | 0.13 | -0.27 | 0.13 |
| incoming_distinctcontacts_std | 0.02 | 0.11 | 0.02 | 0.11 | 0.21 | -0.20 | 0.23 |
| incoming_meanduration_avg | 0.21 | 0.10 | 0.21 | 0.11 | 0.15 | 0.00 | 0.39 |
| incoming_meanduration_std | 0.21 | 0.10 | 0.21 | 0.10 | 0.13 | 0.01 | 0.40 |
| incoming_sumduration_avg | 0.12 | 0.10 | 0.12 | 0.10 | 0.09 | -0.08 | 0.31 |
| incoming_sumduration_std | 0.11 | 0.10 | 0.11 | 0.10 | 0.09 | -0.09 | 0.30 |
| incoming_maxduration_avg | 0.18 | 0.10 | 0.18 | 0.10 | 0.10 | -0.02 | 0.37 |
| incoming_maxduration_std | 0.16 | 0.10 | 0.17 | 0.10 | 0.11 | -0.04 | 0.35 |
| incoming_entropyduration_avg | -0.16 | 0.10 | -0.16 | 0.10 | 0.05 | -0.34 | 0.04 |
| incoming_entropyduration_std | -0.09 | 0.10 | -0.09 | 0.10 | 0.11 | -0.29 | 0.11 |
| outgoing_count_avg | -0.10 | 0.10 | -0.10 | 0.10 | 0.04 | -0.29 | 0.09 |
| outgoing_count_std | -0.08 | 0.10 | -0.08 | 0.10 | 0.04 | -0.27 | 0.11 |
| outgoing_distinctcontacts_avg | -0.06 | 0.10 | -0.06 | 0.10 | 0.07 | -0.25 | 0.14 |
| outgoing_distinctcontacts_std | 0.01 | 0.10 | 0.01 | 0.10 | 0.10 | -0.19 | 0.21 |
| outgoing_meanduration_avg | 0.25 | 0.10 | 0.25 | 0.11 | 0.16 | 0.04 | 0.43 |
| outgoing_meanduration_std | 0.25 | 0.10 | 0.26 | 0.11 | 0.21 | 0.04 | 0.44 |
| outgoing_sumduration_avg | 0.10 | 0.10 | 0.10 | 0.10 | 0.06 | -0.09 | 0.29 |
| outgoing_sumduration_std | 0.12 | 0.10 | 0.12 | 0.10 | 0.09 | -0.08 | 0.31 |
| outgoing_maxduration_avg | 0.14 | 0.10 | 0.14 | 0.10 | 0.12 | -0.07 | 0.33 |
| outgoing_maxduration_std | 0.15 | 0.10 | 0.15 | 0.11 | 0.16 | -0.06 | 0.34 |
| outgoing_entropyduration_avg | -0.13 | 0.10 | -0.13 | 0.10 | 0.10 | -0.32 | 0.07 |
| outgoing_entropyduration_std | -0.10 | 0.10 | -0.10 | 0.10 | 0.12 | -0.30 | 0.11 |

### Location

| feature | r | rse | fisher_r | fisher_rse | fmi | lower95 | upper95 |
| --- | --- | --- | --- | --- | --- | --- | --- |
| stdlengthstayatclusters_avg | 0.06 | 0.11 | 0.06 | 0.11 | 0.17 | -0.15 | 0.26 |
| stdlengthstayatclusters_std | 0.06 | 0.11 | 0.06 | 0.11 | 0.26 | -0.16 | 0.28 |
| circadianmovement_avg | 0.05 | 0.11 | 0.05 | 0.11 | 0.22 | -0.17 | 0.26 |
| circadianmovement_std | -0.01 | 0.12 | -0.01 | 0.12 | 0.28 | -0.23 | 0.22 |
| loglocationvariance_avg | 0.04 | 0.11 | 0.04 | 0.11 | 0.15 | -0.17 | 0.24 |
| loglocationvariance_std | -0.01 | 0.11 | -0.01 | 0.11 | 0.26 | -0.23 | 0.21 |
| movingtostaticratio_avg | 0.11 | 0.11 | 0.11 | 0.11 | 0.23 | -0.10 | 0.32 |
| movingtostaticratio_std | -0.05 | 0.11 | -0.05 | 0.11 | 0.27 | -0.26 | 0.18 |
| locationentropy_avg | 0.01 | 0.11 | 0.01 | 0.11 | 0.27 | -0.22 | 0.23 |
| locationentropy_std | 0.01 | 0.11 | 0.01 | 0.11 | 0.24 | -0.21 | 0.22 |
| locationroutineindex_avg | 0.23 | 0.10 | 0.24 | 0.11 | 0.21 | 0.02 | 0.42 |
| locationroutineindex_std | 0.17 | 0.11 | 0.17 | 0.11 | 0.25 | -0.05 | 0.38 |
| totaldistance_avg | 0.04 | 0.11 | 0.04 | 0.11 | 0.23 | -0.17 | 0.25 |
| totaldistance_std | 0.06 | 0.11 | 0.06 | 0.11 | 0.26 | -0.16 | 0.28 |
| normalizedlocationentropy_avg | 0.01 | 0.11 | 0.01 | 0.11 | 0.27 | -0.21 | 0.23 |
| normalizedlocationentropy_std | -0.03 | 0.13 | -0.03 | 0.13 | 0.44 | -0.28 | 0.22 |
| numberlocationtransitions_avg | -0.03 | 0.10 | -0.03 | 0.10 | 0.10 | -0.23 | 0.17 |
| numberlocationtransitions_std | -0.09 | 0.10 | -0.09 | 0.11 | 0.14 | -0.29 | 0.11 |
| locationvariance_avg | -0.01 | 0.10 | -0.01 | 0.10 | 0.07 | -0.21 | 0.18 |
| locationvariance_std | -0.02 | 0.10 | -0.02 | 0.10 | 0.03 | -0.21 | 0.18 |
| meanlengthstayatclusters_avg | 0.03 | 0.11 | 0.03 | 0.11 | 0.20 | -0.19 | 0.24 |
| meanlengthstayatclusters_std | 0.00 | 0.11 | 0.00 | 0.11 | 0.26 | -0.22 | 0.22 |
| outlierstimepercent_avg | -0.01 | 0.10 | -0.01 | 0.10 | 0.13 | -0.21 | 0.20 |
| outlierstimepercent_std | 0.00 | 0.11 | 0.00 | 0.11 | 0.24 | -0.22 | 0.21 |
| maxlengthstayatclusters_avg | 0.06 | 0.10 | 0.06 | 0.10 | 0.08 | -0.14 | 0.25 |
| maxlengthstayatclusters_std | 0.03 | 0.11 | 0.03 | 0.11 | 0.15 | -0.17 | 0.24 |
| numberofsignificantplaces_avg | -0.01 | 0.10 | -0.01 | 0.10 | 0.10 | -0.21 | 0.19 |
| numberofsignificantplaces_std | -0.01 | 0.11 | -0.01 | 0.11 | 0.13 | -0.22 | 0.19 |
| timeattop1_avg | 0.12 | 0.10 | 0.12 | 0.10 | 0.10 | -0.08 | 0.31 |
| timeattop1_std | 0.11 | 0.10 | 0.11 | 0.11 | 0.13 | -0.10 | 0.31 |
| timeattop2_avg | 0.05 | 0.11 | 0.05 | 0.11 | 0.17 | -0.16 | 0.25 |
| timeattop2_std | 0.02 | 0.12 | 0.02 | 0.12 | 0.31 | -0.21 | 0.25 |
| timeattop3_avg | 0.24 | 0.26 | 0.25 | 0.27 | 0.88 | -0.28 | 0.66 |
| timeattop3_std | 0.18 | 0.25 | 0.19 | 0.26 | 0.86 | -0.30 | 0.60 |

### Screen

| feature | r | rse | fisher_r | fisher_rse | fmi | lower95 | upper95 |
| --- | --- | --- | --- | --- | --- | --- | --- |
| screen_countepisode_avg | 0.00 | 0.10 | 0.00 | 0.10 | 0.00 | -0.19 | 0.19 |
| screen_countepisode_std | -0.06 | 0.10 | -0.06 | 0.10 | 0.00 | -0.25 | 0.13 |
| screen_sumduration_avg | 0.23 | 0.09 | 0.23 | 0.10 | 0.00 | 0.04 | 0.40 |
| screen_sumduration_std | 0.17 | 0.10 | 0.17 | 0.10 | 0.00 | -0.02 | 0.35 |
| screen_maxduration_avg | 0.24 | 0.09 | 0.24 | 0.10 | 0.00 | 0.05 | 0.41 |
| screen_maxduration_std | 0.17 | 0.10 | 0.17 | 0.10 | 0.00 | -0.02 | 0.35 |
| screen_avgduration_avg | 0.37 | 0.08 | 0.39 | 0.10 | 0.00 | 0.20 | 0.53 |
| screen_avgduration_std | 0.27 | 0.09 | 0.28 | 0.10 | 0.00 | 0.09 | 0.44 |
| screen_ri_alldays_avg_avg | 0.00 | 0.10 | 0.00 | 0.10 | 0.00 | -0.19 | 0.19 |
| screen_ri_alldays_avg_std | 0.00 | 0.10 | 0.00 | 0.10 | 0.00 | -0.19 | 0.19 |
| screen_ri_alldays_range_avg | 0.01 | 0.10 | 0.01 | 0.10 | 0.00 | -0.18 | 0.20 |
| screen_ri_alldays_range_std | 0.01 | 0.10 | 0.01 | 0.10 | 0.00 | -0.18 | 0.20 |
| screen_entropy_avg | 0.08 | 0.10 | 0.08 | 0.10 | 0.00 | -0.11 | 0.26 |
| screen_entropy_std | -0.09 | 0.10 | -0.09 | 0.10 | 0.00 | -0.27 | 0.11 |
| screen_normalisedEntropy_avg | 0.08 | 0.10 | 0.08 | 0.10 | 0.00 | -0.11 | 0.27 |
| screen_normalisedEntropy_std | -0.11 | 0.10 | -0.11 | 0.10 | 0.00 | -0.29 | 0.08 |
